# Development of Cyclic Peptides Targeting the Epidermal Growth Factor Receptor in Mesenchymal Triple-Negative Breast Cancer Subtype

**DOI:** 10.3390/cells12071078

**Published:** 2023-04-03

**Authors:** Nancy Nisticò, Annamaria Aloisio, Antonio Lupia, Anna Maria Zimbo, Selena Mimmi, Domenico Maisano, Rossella Russo, Fabiola Marino, Mariangela Scalise, Emanuela Chiarella, Teresa Mancuso, Giuseppe Fiume, Daniela Omodei, Antonella Zannetti, Giuliana Salvatore, Ileana Quinto, Enrico Iaccino

**Affiliations:** 1 Department of Experimental and Clinical Medicine, Magna Graecia University of Catanzaro, 88100 Catanzaro, Italy; 2 Department of Life and Environmental Sciences, University of Cagliari, Cittadella Universitaria di Monserrato, Monserrato, 09042 Cagliari, Italy; 3 Net4Science srl, University “Magna Græcia”, 88100 Catanzaro, Italy; 4 Department of Medical Oncology, Jerome Lipper Multiple Myeloma Center, Dana-Farber Cancer Institute, Harvard Medical School, Boston, MA 02215, USA; 5 Department of Pharmacy, Health and Nutritional Sciences, University of Calabria, 87036 Rende, Italy; 6 “Annunziata” Regional Hospital Cosenza, 87100 Cosenza, Italy; 7 Institute of Biostructures and Bioimaging, National Research Council, IBB-CNR, 80145 Naples, Italy; 8 Dipartimento di Scienze Motorie e del Benessere, Università degli studi di Napoli “Parthenope”, 80133 Naples, Italy; 9 CEINGE- Biotecnologie Avanzate S.C.A.R.L., 80145 Naples, Italy

**Keywords:** EGFR, triple-negative breast cancer, breast cancer biomarkers, peptide, phage display, tumor targeting

## Abstract

Triple-negative breast cancer (TNBC) is an aggressive malignancy characterized by the lack of expression of estrogen and progesterone receptors and amplification of human epidermal growth factor receptor 2 (HER2). Being the Epidermal Growth Factor Receptor (EGFR) highly expressed in mesenchymal TNBC and correlated with aggressive growth behavior, it represents an ideal target for anticancer drugs. Here, we have applied the phage display for selecting two highly specific peptide ligands for targeting the EGFR overexpressed in MDA-MB-231 cells, a human TNBC cell line. Molecular docking predicted the peptide-binding affinities and sites in the extracellular domain of EGFR. The binding of the FITC-conjugated peptides to human and murine TNBC cells was validated by flow cytometry. Confocal microscopy confirmed the peptide binding specificity to EGFR-positive MDA-MB-231 tumor xenograft tissues and their co-localization with the membrane EGFR. Further, the peptide stimulation did not affect the cell cycle of TNBC cells, which is of interest for their utility for tumor targeting. Our data indicate that these novel peptides are highly specific ligands for the EGFR overexpressed in TNBC cells, and thus they could be used in conjugation with nanoparticles for tumor-targeted delivery of anticancer drugs.

## 1. Introduction

Triple-negative breast cancer (TNBC) is the most aggressive subtype accounting for 15–20% of worldwide-diagnosed breast tumors [1]. TNBC cells are characterized by the lack of expression of estrogen (ER) and progesterone receptors (PR) and the human epidermal growth factor receptor 2 (HER2) amplification. Thus, in clinical practice, the diagnosis of TNBC is based on immunohistochemical staining and imaging procedures [2,3,4]. TNBC is a highly proliferative and invasive tumor associated with a high risk of relapse, poor prognosis, and limited treatment options [5,6]. This aggressive behavior is likely due to the high intra-tumor heterogeneity. Indeed, Lehmann et al. identified six different molecular subcategories within the clinical subtype of TNBC by transcriptomic analysis [7], which were subsequently refined in four clinically relevant TNBC subtypes: basal-like (BL) 1, BL-2, mesenchymal (M), and immunomodulatory (IM) status [8,9,10].

Treatment options for TNBC patients mainly include surgery and chemotherapy [11]. Unfortunately, TNBC patients frequently develop acquired drug resistance, associated with a high risk of recurrence and the spread of metastasis, making this tumor intractable. Many drug resistance mechanisms are not yet well understood, and several targeted therapies are currently under pre- and clinical investigation [12,13,14,15,16]. Finding novel targetable molecules offers opportunities for the development of therapeutic strategies, which are able to improve the efficacy of the treatment and patient survival compared to standard chemotherapy alone. In this context, the epidermal growth factor receptor (EGFR) is a central player in the progression of many solid tumors, and it is upregulated in more than half of the TNBC cases [17]. EGFR was found to be enriched in the mesenchymal TNBC subtype, where a high level of mRNA expression was associated with the poor prognosis of TNBC patients and failure of conventional therapies [18,19,20]. EGFR is a tyrosine kinase transmembrane glycoprotein that belongs to the ErbB family [21]. It has an extracellular region (EC) consisting of ~620 amino acids divided into four domains (I–IV), a transmembrane (TM) region, and an intracellular cytoplasmic receptor tyrosine kinase (TK) region. The Epidermal Growth Factor (EGF) ligand binds to the EC of EGFR, inducing a conformational change that leads to the homo- or hetero-oligomerization of the receptor tyrosine kinase and the activation signaling of cell proliferation and differentiation [22]. The same binding region is the docking site for other different ligands, peptides, or proteins, which simultaneously initiate a series of signaling cascades to produce a physiological or pathological outcome. The aberrant activation of the EGFR downstream signaling can promote tumorigenesis [23]. Therefore, EGFR-targeted therapies based on monoclonal antibodies (mAb) [24] and tyrosine kinase inhibitors (TKI) have been implemented in the last two decades to improve the treatment of solid tumors overexpressing EGFR [25,26]. However, these drugs have not been effective in patients with TNBC, and new experimental approaches have been directed toward the development of drug conjugates with antibodies or peptides targeting EGFR. Interestingly, peptide ligands of EGFR could be conjugated to nano-vehicles carrying anticancer drugs to implement TNBC-targeted therapy [27].

In this view, the phage display technique allows a rapid selection of peptide ligands for membrane receptors, such as EGFR overexpressed on tumor cells, as an alternative and powerful tool for the development of anticancer strategies [28]. The phage display method consists of the selection of peptide-expressed phage clones from highly diversified peptide libraries (>10^9^ different peptide sequences), which have high specificity and affinity binding for a target [29,30]. The screening process is called “biopanning” and consists of the selection of bacteriophages during sequential receptor binding steps under conditions of increasing stringency followed by clone amplification. After 3–5 cycles of affinity-driven biopanning, the DNA insert of the selected phage ligands is sequenced to determine the amino acid primary structure of the visualized peptides [28]. Linear (lPep) or cyclic peptides (cPep) can mimic natural ligands and act in an antagonistic or agonistic manner, and they are also suitable for the structural analysis of protein-protein interaction (PPi) [31]. Peptide drug delivery holds great promise in pharmaceutical research [32]. In this regard, in silico computational methods can help to rationally design lPep and cPep, making them a fascinating therapeutic approach that guarantees specificity and tolerability [33]. However, it is also true that lPep or cPep have drug limitations (administration, poor pharmacokinetic profile, bioavailability) but can be modified to create conformationally bound analogs and peptidomimetics, with an enhanced ADME profile, taking into account the common characteristics of the chemical pharmacophore [34,35,36]. In the last decades, several research groups have been focused on EGFR and the identification of specific peptide ligands with biological activity [37,38,39,40,41,42,43,44,45]. However, in these studies, authors used the human recombinant protein EGFR for phage display screening in vitro with phage display libraries composed of linear 7-mer or 12-mer peptides. The chosen peptide library represented a limitation in these previous studies because linear peptides possess lower structural rigidity and stability in solution, and therefore they need to be chemically modified to improve the binding efficiency in vitro and in vivo. In this study, we have screened a phage display peptide library that exposes a seven amino acid insert flanked by disulphide-linked cysteines. The peptides are smaller in size, and the closed loop ensures the rigidity of the peptide structure, allowing the amino acid sequence to position itself correctly with respect to the target epitope. Furthermore, we have performed an in vitro screening directly on TNBC cell lines, enforcing the binding validations of our protocol. Our experimental approach allowed us to isolate two stable peptides with high affinity and specificity binding for the native form of EGFR. In perspective, these peptides could be used to decorate drug delivery systems, such as peptide-drug conjugated nanoparticles, in order to target EGFR-positive tumor cells.

## 2. Materials and Methods

### 2.1. Cell Lines and Culture Conditions

Human TNBC cell lines (wild type and CRISPR-CaS9—EGFR-silenced MDA-MB-231 [46] and BT-549), 4T1 murine mammary carcinoma cells, and human B cell lymphoma Ramos cell line were cultured in RPMI-1640 medium (Life Technologies) supplemented with 10% heat-inactivated fetal bovine serum (FBS) (Thermo Fisher Scientific, Waltham, MA, USA).

K1 (thyroid papillary carcinoma) and Cal-62 (thyroid gland anaplastic carcinoma) cell lines were cultured in Dulbecco’s Modified Eagle’s Medium (DMEM) (Thermo Fisher Scientific) containing 10% FBS. All cell types were grown in 100 U/mL penicillin and 100 μg/mL streptomycin (Thermo Fisher Scientific) in a humidified atmosphere, 5% CO_2_, at 37 °C.

### 2.2. Phage Display Screening

The Ph.D.™-C7C Phage Display Peptide Library Kit (#E8120S—New England Biolabs, Ipswich, MA, USA) is a combinatorial library of random peptides with a disulfide-constrained loop, with the displayed peptides expressed as AC-X7-CGGGS fused at the N-terminus of the minor coat pIII of M13 phage. This library was used to screen phage ligands to the EGFR overexpressed in the TNBC cell line MDA-MB-231. As a first step, we plated EGFR-silenced (EGFR KO) and wild-type MDA-MB-231 cells at the same concentration in a 6-well plate (5 × 10^5^ cells/well). After cell adhesion, we pre-incubated 10 μL of the phage library (~1 × 10^13^ pfu/mL) with MDA-MB-231 EGFR-KO cells for 2 h at 37 °C to remove non-specific bound phages. Then, the supernatant containing the unbound phages was separated from the cell debris by centrifugation and incubated with wild-type MDA-MB-231 cells overexpressing EGFR for 1 h at 37 °C. An increasing number of washes with PBS 0.1% BSA was performed in the subsequent three screening rounds to eliminate unbound phages (1, 3, and 5 fold, respectively). After each round of selection, eluted phages were titred to determine phage enrichment (number of pfu/mL). *Escherichia coli* strain ER2738 K12 (NEB) was used for the amplification of M13 phage in LB Broth (Sigma-Aldrich Corp., St. Louis, MO, USA) following the manufacturer’s instructions (NEB). After three rounds of phage selection and amplification, individual phage clones were randomly selected and amplified to extract genomic DNA.

### 2.3. Phage DNA Purification and Sequencing

Single-stranded DNA was extracted from the selected phage clones using an equal volume of 25:24:1 phenol/chloroform/isoamyl alcohol (Sigma-Aldrich) followed by centrifugation for 15 min at 16,000× *g.* The aqueous phase was precipitated with 0.8 volumes of absolute ethanol and 0.2 volumes of 3M sodium acetate (pH 4.6) and incubated for 1 h at −80 °C. After centrifugation for 15 min at 16,000× *g*, the pellet was washed with 70% ethanol and centrifuged for 30 min at 16,000× *g*. DNA was dissolved in nuclease-free water, visualized by agarose gel electrophoresis, and sent to custom DNA sequencing (Eurofins Genomics). The nucleotide sequences encoding the random peptide insert were analyzed by an online bioinformatics tool (http://bio.biomedicine.gu.se/edu/translat.html, accessed on 24 November 2021) to determine their primary amino acid sequence.

### 2.4. FITC Phages Labeling Kit

Phages expressing peptide sequences (01cys_EGFR, 04cys_EGFR, 06cys_EGFR, and 30cys_EGFR) and a wild-type M13 phage were conjugated to fluorescein isothiocyanate (FITC) in order to analyze their binding affinity to EGFR on MDA-MB-231 by flow cytometry. The “Pierce FITC Antibody Labeling Kit” (cat #53027—Thermo Fisher Scientific) was used for the conjugation following the manufacturer’s instructions.

### 2.5. Bioinformatics Analysis of Peptide Conformation

The 3D structures of both 01cys_EGFR (Cys-EKMVATH-Cys) and 06cys_EGFR (Cys-PSDEHHT-Cys) peptides were built by using the Maestro GUI (Software: Maestro Schrodinger, LLC, New York, NY, USA. 2018-3) and submitted to Schrodinger’s Protein Preparation Wizard (charges and the Optimized Potentials for Liquid Simulations-all atom (OPLS-AA) force field 2005 parameters) [47]. To investigate the conformational variability of peptides, 200 ns of Molecular Dynamics simulations (MDs) using the Desmond package [48] were run. MDs were conducted in the isothermal-isobaric ensemble at 300 K under 1 atm pressure. Transferable intermolecular potential with 3 points (TIP3P) [49] explicit solvation model was adopted for considering the water solvent effects on peptides conformational properties and OPLS2005 [47] as the force field. The trajectory coordinates were submitted to clustering analysis, and for each peptide, the best representative conformation of the best cluster was docked into the binding region of EGFR. In particular, seven (sizes: 401, 284, 116, 68, 65, 41, 27) and six clusters (sizes: 307, 279, 176, 138, 63, 39) were generated for cPep-01 and cPep-06, respectively.

### 2.6. Peptides-EGFR Docking Studies

HADDOCK is a docking tool for bimolecular complexes modeling that combines Coulomb electrostatic energies, unbonded intermolecular Van der Waals, and empirically derived desolvation energies buried surface area [50,51]. The sampling parameters were followed for each representative structure of the two peptides: 1000 structures for rigid-body docking, 200 structures for the final refinement, and a cut-off equal to 5.0 to define neighboring flexible regions. Ten clusters were examined with their top 4 docked structures. The clusters were numbered based on their size and sorted based on the HADDOCK score; the Z-score indicates how many standard deviations from the average this cluster is located in terms of score (the more negative, the better). Further, scoring is a weighted sum of van der Waals, electrostatic, desolvation, and restraint violation energies with the buried surface area. To improve the analysis and predict the binding affinity ΔG (kcal mol^−1^) of 01cys_EGFR and 06cys_EGFR in the EGFR site, the two complexes were submitted to the protein binding energy prediction (PRODIGY) server [52,53]. The stability of the peptide-protein complex was measured through the dissociation constant Kd (M) at 37 °C. Schrodinger’s Maestro visualization was used to visualize the structures Software: Maestro Schrodinger, LLC, New York, NY, USA. 2018-3 [54]. LigandScout 4.3 was used to visualize the key pharmacophoric features in the two complexes [55].

### 2.7. Peptide Synthesis

Peptides 01_cysEGFR and 06_cys EGFR were custom synthesized by Caslo Laboratory ApS (Kongens Lyngby, Denmark). The irrelevant peptide (CGGNGPGLC) was synthesized as a negative control. For binding assays, we used synthetic peptides that were FITC-conjugated at the N-terminus via aminohexanoic acid (Ahx) linker, amidated at the C-terminus and circularized by one disulphide bond between the two cysteines flanking the peptide sequence. For biological assays, free soluble peptides were produced with the following modifications: one disulphide bond and an amidated C-terminus.

### 2.8. Western Blotting

Total protein extracts were prepared from 1–4 × 10^6^ cells suspended in RIPA buffer (Tris-HCl 50 mM, NaCl 150 mM, 1% Nonidet P-40, 0.5% sodium deoxycholate, 0.1% SDS, freshly added 1% protease inhibitor and phosphatase inhibitors), incubated for 30 min on ice, and then subjected to three cycles of freezing and thawing. Cell lysates were centrifuged at 14,000× *g* for 20 min, and the supernatant containing total protein extracts was harvested for use. Total protein concentrations were quantified using Bio-Rad Protein Assay Dye Reagent Concentrate, and absorbance values were compared to a BSA standard curve. Proteins (25 μg) were diluted in 1× NuPAGE sample buffer and 1× Reducing buffer, boiled at 70 °C for 10 min, and separated by electrophoresis on 4–12% NuPAGE Novex bis-Tris gradient polyacrylamide gels (Thermo Fisher Scientific). Proteins were transferred onto a nitrocellulose membrane of 0.45 μm (Biorad). Membranes were blocked at room temperature in a blocking solution (5% non-fat dry milk in Tween-Tris Buffered Saline 1×) for 1 h. The primary antibodies, purchased from Cell Signaling Technology (Danvers, MA, USA), were diluted in blocking solution and incubated overnight at 4 °C: rabbit monoclonal EGFR (Cat# 4267 Cell Signaling Technology, Danvers, MA, USA) at dilution 1:1000 and mouse monoclonal anti-GAPDH (sc-47724 Santa Cruz Biotechnology, Dallas, TX, USA) at 1:3000 dilution. Membranes were incubated with secondary antibodies anti-rabbit IgG (NA934-1ML; GE Healthcare Life Sciences, Marlborough, MA, USA) and anti-mouse IgG (NXA931-1ML; GE Healthcare Life Sciences Marlborough, MA, USA) conjugated with horseradish peroxidase. Proteins on membrane filters were visualized using the ECL Western Blotting Substrate (W1001-Promega, Madison, WI, USA) and acquired on the Alliance Mini HD9 system (UVITEC Ltd., Cambridge, UK).

### 2.9. Cell Binding Assay

We analyzed the binding of FITC-conjugated phages on MDA-MB231 cells by flow cytometry. A binding assay was also performed with FITC-conjugated peptides to human MDA-MB-231 and BT-549 cells, murine 4T1 cells, and additional EGFR-positive thyroid cancer cell lines K1 and Cal62. To increase the stringency conditions of in vitro binding, adherent cells were incubated with 10 μg/mL of 01cys_EGFR and 06_cys EGFR for 45 min in the dark at 37 °C instead of 4 °C of the standard protocol. Cells were recovered by a cell scraper, then twice washed with PBS and suspended in 300 μL of BD™ FACSFlow™ fluid. Data were acquired on the BD FACSCanto™ II and analyzed with FlowJo software (Becton Dickinson, San Jose, CA, USA).

### 2.10. Cell Cycle Analysis

BT549 cells (200,000 cells/well) were seeded onto a 6-well plate in RPMI-1640 complete medium. After adhesion, cells were starved in serum-free RPMI-1640 for 18 h, and then the medium was changed to RPMI-1640 supplemented with 0.2% FBS in the presence or absence of control, 01cys_EGFR and 06_cys EGFR peptides (25 μM). After 24 h of peptide stimulation, cells were trypsinized, washed, and suspended in PBS. Cells were fixed with 1 mL of ice-cold 70% ethanol and incubated for 30 min at 4 °C. Fixed cells were washed three times and stained with 50 μg/mL of propidium iodide and 40 μg/mL of RNAse A. Cell cycle profile was acquired by FACS Canto II and analyzed by Flow Jo software 8.8.6 (Becton Dickinson).

### 2.11. Immunofluorescence

OCT-embedded MDA-MB-231 xenograft tissue samples derived from Balb/c nude mice were cut in 8–10 µm sections and mounted onto microscope slides for confocal microscopy analysis. Sections were fixed with 4% paraformaldehyde for 30 min at 4 °C, and then for 45 min, incubated at 4 °C with rabbit polyclonal anti-EGFR (1:100 dilution -Bethyl Laboratories, Inc., Montgomery, TX, USA) followed by Alexa Fluor 568-labeled anti-rabbit secondary antibody (1:500 dilution; Thermo Fisher Scientific). After washing, cells were incubated in the dark with FITC-conjugated peptides (Control, 01_cysEGFR, 06_cysEGFR) (10 μg/mL) for 45 min at 37 °C, anti-Wheat Germ Agglutinin (WGA) Alexa Fluor 647 conjugate (1:200 dilution; Thermo Fisher Scientific) for 45 min, and finally with DAPI at the dilution 1:500 for 10 min. Cells were prepared with mounting medium (ProLong antifade, Thermo Fisher Scientific) and visualized by confocal microscopy (LEICA TCS SP8).

### 2.12. Statistical Analysis

Data are presented as mean ± standard deviation from at least two independent experiments. Statistical analysis was performed with a Student *t*-test (* *p* < 0.05, ** *p* < 0.005, *** *p* < 0.0001).

## 3. Results

### 3.1. Selection and Identification of EGFR Binding Peptides by Phage Display

**Figure 1:**
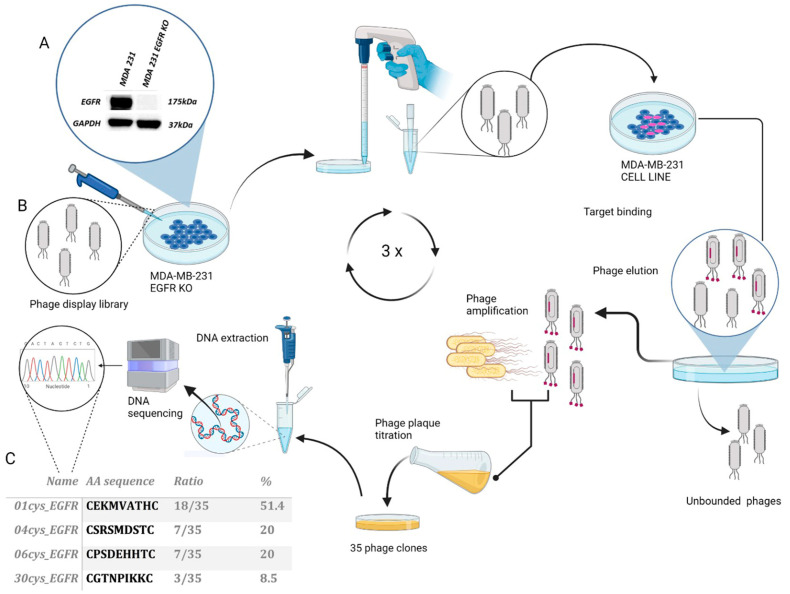
Selection and identification of peptide ligands for EGFR in the TNBC cell line MDA-MB231. Schematic representation of phage display screening: (**A**) Protein expression levels of EGFR in MDA-MB-231 wild-type and EGFR KO cells were verified by Western blot analysis; GAPDH expression was included as loading control. (**B**) Non-specifically bound phages were eliminated by incubation of the library with MDA-MB-231 EGFR KO cells. Unbound phages were then incubated with MDA-MB-231 overexpressing EGFR to select phages with high affinity binding. Three cycles of selection and amplification were performed with increasing stringency conditions. The genomic DNA of selected phage clones was extracted and sequenced to determine the peptide insert required for EGFR binding. (**C**) Peptide sequence and occurrence frequency of selected phage clones.

For screening the phage peptide library, we used as bait the MDA-MB-231 cell line, a mesenchymal TNBC subtype over-expressing EGFR. Initially, we assessed the expression level of EGFR protein in MDA-MB-231 cells, wild-type and CRISPR-silenced for EGFR (EGFR KO), by immunoblot of total protein lysates. The EGFR protein of 175KDa was detected in wild-type MDA-MB-231 while undetected in MDA-MB-231 EGFR KO cells (Figure 1A). Subsequently, we performed phage display screening by using a cyclic combinatorial peptide library in C7C format produced in phage M13 (Figure 1B). We first screened the phage library with MDA-MB-231 EGFR KO cells as bait in order to eliminate from the library the phages that bound to the membrane cells in the absence of EGFR (i.e., non-specific binding). Then, the unbound phages were incubated with MDA-MB-231 cells overexpressing EGFR. To isolate phages with higher affinity to EGFR, increasing washes with PBS 0.1% BSA were performed in the subsequent three screening rounds to eliminate unbound phages (1, 3, and 5 fold, respectively). The bound phages were eluted, amplified, and titred. After three sequential rounds of selection, a progressive reduction in the number of phage ligands was observed in relation to conditions of greater stringency (Supplementary Figure S1). From the eluate of the third round of selection, 35 individual phage plaques were randomly selected for amplification. The phage DNA was purified, sequenced, and analyzed to determine the amino acid sequence corresponding to the random peptide insert in the phage membrane. Based on this analysis, four peptide sequences were identified: 01cys_EGFR (CEKMVATHC), 04cys_EGFR (CSRSMDSTC), 06cys_EGFR (CPSDEHHTC), and 30cys_EGFR (CGTNPIKKC) (Figure 1C).

The binding ability of phages 01cys_EGFR, 04cys_EGFR, 06cys_EGFR, and 30cys_EGFR to the MDA-MB-231 cells were analyzed by flow cytometry. To this end, the phages were conjugated with fluorescein isothiocyanate (FITC) and incubated with wild-type MDA-MB-231 or MDA-MB-231 EGFR- KO cells. The FITC-conjugated wild-type phage was used as a negative control. As shown in Supplementary Figure S2, the phages 01cys_EGFR and 06cys_EGFR bound to wild-type MDA-MB-231 cells with higher efficiency compared to 04cys_EGFR and 30cys_EGFR phages as measured by the percentage of fluorescence. Consistently, MDA-MB-231 EGFR-KO remained unstained, confirming the absence of non-specific bacteriophage binding. Based on these results, we considered the phages 01cys_EGFR and 06cys_EGFR as the best candidates for further investigation.

### 3.2. 01_cys EGFR and 06_cys EGFR Docking Studies in the EGFR Pocket

**Figure 2:**
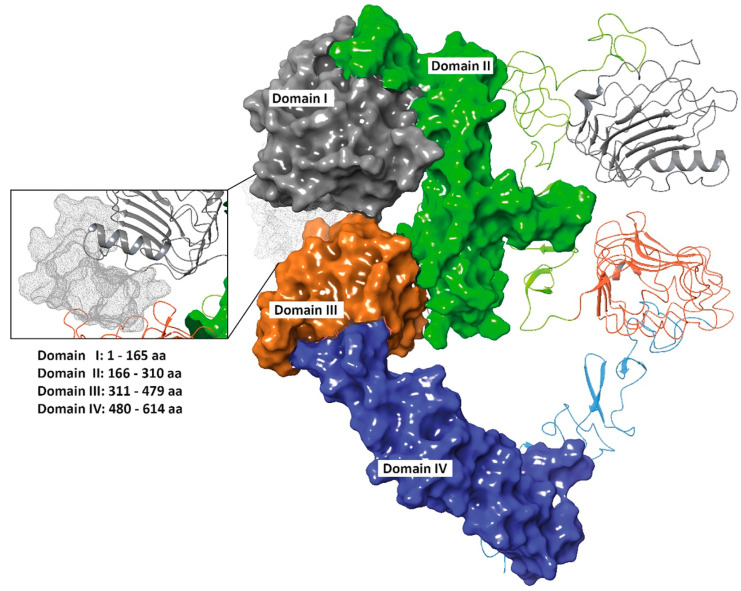
Three-dimensional front view of EGFR. Domain I (1–165 amino acid residues), II (166–310 amino acid residues), III (311–79 amino acid residues), and IV (480–614 amino acid residues) are colored in grey, green, orange and blue, respectively. The cavity region between Domain I and III in the EC region is highlighted in the lateral popup and colored in grey dots (PDB code: 3NJP).

The EGFR binding site was determined from the EGF-EGFR complex, retrieved from the Protein Data Bank (PDB code 3NJP [56]) (Figure 2A) and chosen to perform peptide-protein recognition of 01_cys EGFR and 06_cys EGFR (amino acid residues: 12–18, 29, 90–100, 346–360, 384, 409, 465–468).

**Figure 3:**
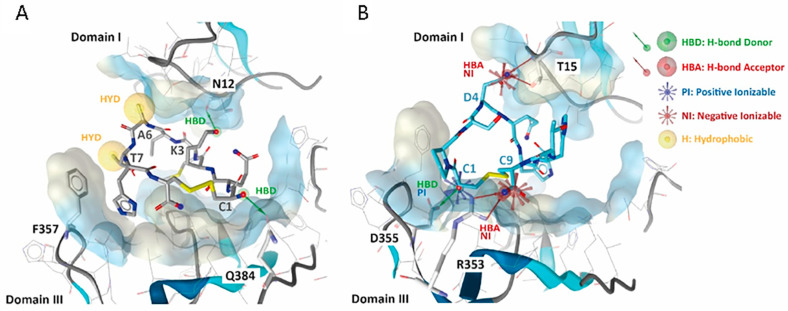
Principal amino acid (aa) interactions involved in the binding site of EGFR between 01_cys EGFR (**A**) and 06_cys EGFR (**B**) are labeled and highlighted in the stick model. On the right of the figure, the chemical pharmacophore features are explained. The EGFR cavity is shown in solid style.

Several molecular modeling methods and bioinformatics tools dedicated to peptide-protein binding affinity prediction have been recently developed [57,58,59,60,61,62]. We used High Ambiguity Driven protein-protein DOCKing (HADDOCK) vers. 2.4 for docking simulations [50]. Results revealed that Van der Waals (VdW) interactions in the 01_cys EGFR (−37.5 ± 2.2 kcal/mol) complex are better than 06_cys EGFR (−33.2 ± 3.5 kcal/mol) complex. Vice versa, the electrostatic interactions for 06_cys EGFR (−198.4 ± 42.3 kcal/mol) complex were found to be better than 01_cys EGFR (−111.7 ± 22.3 kcal/mol) complex. Docking analysis in the EGFR pocket showed that 01_cys EGFR established two H-bonding donors (HBD) with N12 and Q384 residues; one remarkable hydrophobic (HYD) interaction with F357, with a total Buried Surface Area (BuSA) of 1176.4 ± 56.0 and a HADDOCK score equal to −42.4 (Figure 3A). BuSA was calculated by taking the difference between the sum of the solvent-accessible surface area for each molecule separately and the solvent-accessible area of the complex. Concerning 06_cys EGFR, we noted two negative (N) and one positive (P) ionizable interactions with T15, R353, and D355 amino acid residues, respectively, followed by as many hydrogen bonds (HBs) (Figure 3B). In both cases, many contacts (39 for 01_cys EGFR and 41 for 06_cys EGFR) occurred between our peptides-binders and the most relevant and well-documented amino acid residues located at the two domains I and III [63]. Related to this point, it is necessary to underline that these chemical forces (VdW, hydrophobic attraction, and ionic interaction) play a critical role in stabilizing PP complexes besides the corresponding BuSA. To better predict the binding affinity and stability—dissociation constant Kd (M) at 37 °C—01_cys EGFR and 06_cys EGFR complexes were submitted to the protein binding energy prediction (PRODIGY) server [52,53]. The binding affinity values were −10.1 kcal mol^−1^ and −8.5 kcal mol^−1^ for the cPep-01-EGFr and cPep-06-EGFr complexes, respectively; the predicted dissociation constant (KD) was 7.7 × 10^−8^ for the cPep-01-EGFr and 1.1 × 10^−6^ for the cPep-06-EGFr complex (Table 1).

### 3.3. Detection of EGFR Protein Expression in Human and Murine TNBC Cell Lines

**Figure 4:**
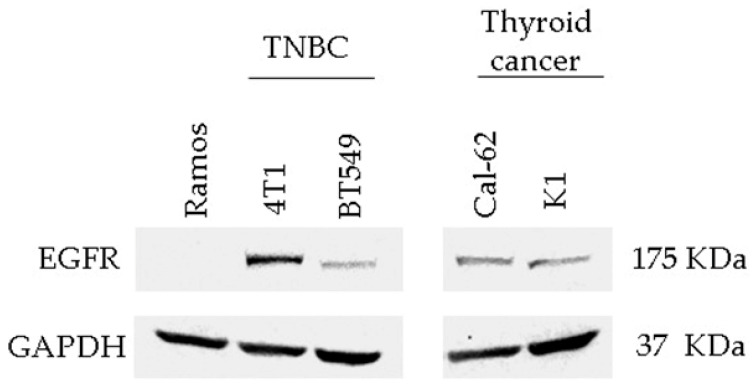
Expression of the EGFR protein in Ramos cells, murine 4T1, human BT-549 TNBC cell lines, and thyroid cancer cell lines K1 and Cal62. Total cell extracts were separated by SDS-PAGE and analyzed by Western blotting with anti-EGFR and anti-GAPDH antibodies.

For the EGFR binding validation assay of peptides, we used the human TNBC cell line BT-549 and the murine TNBC cell line 4T1 cells, which both expressed EGFR even though at different levels; as a negative control, we used Ramos cells, a B lymphocyte cell line negative for EGFR expression (Figure 4). In addition, we measured EGFR protein expression also in thyroid carcinoma cells as other EGFR-positive tumor types for binding validation (Figure 4).

### 3.4. FITC-Conjugate Peptide Binding to Breast Cancer Cell Lines

**Figure 5:**
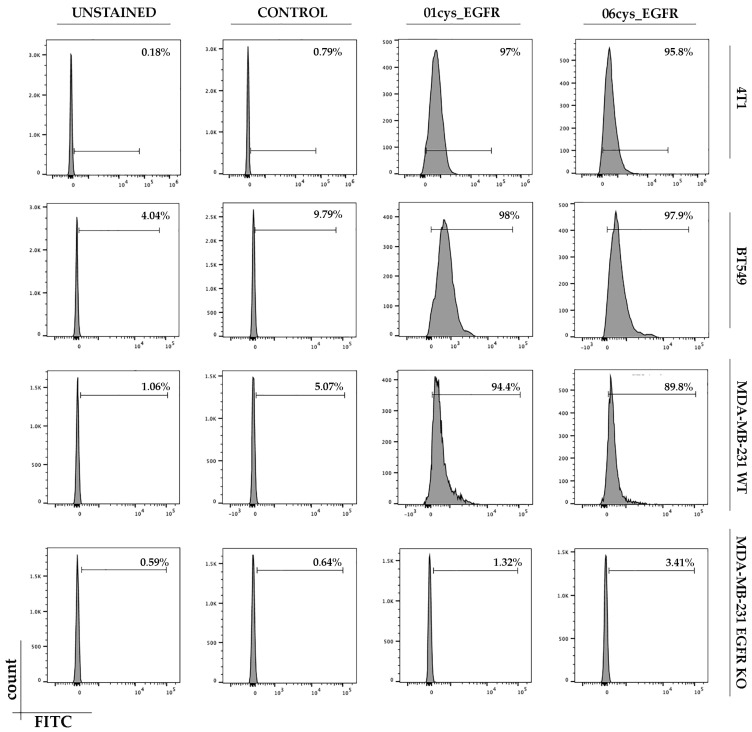
Binding assay of peptides to TNBC cells. Cells were incubated with 01cys_EGFR, 06cys_EGFR peptides, or the irrelevant peptide as control and then analyzed by flow cytometry. Percentage of binding efficiency of peptides is shown in each panel. The TNBC cell lines were: MDA-MB-231, BT-549, and 4T1. The cell line MDA-MB-231 EGFR-KO was included as a negative control.

Based on the percentage of clonal identity and molecular docking results, we first verified the binding ability of the peptides 01_cys EGFR and 06_cys EGFR to MDA-MB-231, MDA-MB-231 EGFR KO, BT549, and 4T1 cells in vitro. Peptide sequences were chemically synthesized as FITC-labeled soluble peptides and were subsequently tested for binding capabilities and specificity outside the phage context. TNBC cells were treated with 10 µM of control, 01_cys EGFR, and 06_cys EGFR peptides. Flow cytometric analysis showed that 01cys_EGFR and 06cys_EGFR peptides were able to bind the human and murine TNBC cells with high efficiency, while they did not bind MDA-MB-231 EGFR KO cells, indicating that the peptide binding to the cells required the presence of membrane EGFR (Figure 5). Further, the irrelevant peptide (CGGNGPGLC), used as a negative control, did not bind any cell type (Figure 5). These results indicated that 01_cys EGFR and 06_cys EGFR peptides were specific ligands of EGFR.

In order to evaluate the binding efficacy of our peptides in other EGFR-overexpressing cancer cell types, we choose two different thyroid cancer cell lines (Cal62 and K1). Flow cytometric analysis performed after peptide incubation indicated a higher efficacy of binding for 01cys_EGFR with respect to 06cys_EGFR peptide, especially in the K1 cells (Supplementary Figure S3).

### 3.5. Ex Vivo EGFR-Specific Targeting of 01_cysEGFR and 06_cysEGFR Peptides

**Figure 6:**
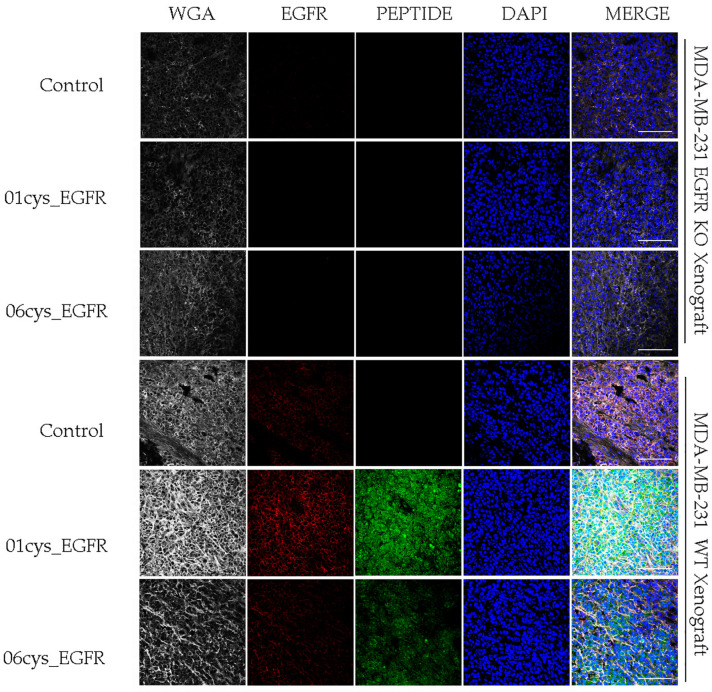
Immunostaining of MDA-MB-231 tumor xenografts with 01_cysEGFR and 06_cysEGFR peptides. Representative confocal images were acquired from MDA-MB-231 tumor xenograft tissue sections stained with anti-EGFR (red), the peptides irrelevant control, 01_cysEGFR and 06_cysEGFR (green), anti-WGA (white) and DAPI (blue). The merge of these channels confirmed the co-localization of EGFR and peptides on the cell membrane. Images were acquired at magnification 40× using a confocal microscope (Leica TC-SP2 Confocal System, Leica Microsystem Srl, Milan, Italy). Scale bar = 100 μm. Representative images of multiple serial sections are shown.

Then, we asked whether the 01_cysEGFR and 06_cysEGFR peptides could be used for in vivo analysis of tumor tissues expressing EGFR. To this end, we generated MDA-MB-231 tumor xenograft in mice and performed confocal microscopy analysis of tumor tissue sections upon immunofluorescence staining with FITC-peptides (green signal) or anti-EGFR (red signal) (Figure 6). Tissue structure and cellular density were inhomogeneous throughout the specimens, with areas that were more preserved compared to others where the extracellular matrix appeared unstructured. 

EGFR was detected in the MDA-MB-231 xenograft tissue sections by an anti-EGFR antibody as a red fluorescence signal, while it was undetected in MDA-MB-231 EGFR KO (Figure 6). Coherently with the data reported before, compared with the 06_cysEGFR, the staining with the peptides 01_cysEGFR gave a stronger green fluorescence signal for MDA-MB-231 xenograft tissue and lack of fluorescence for MDA-MB-231 EGFR KO xenograft, indicating that the presence of EGFR was required for the binding of the two peptides. A lack of green fluorescence signal was observed for the irrelevant control peptide. Consistently, the merged images showed the overlapping of the red signal (EGFR) and green signal (01_cysEGFR) as a result of the co-localization of peptide ligands with EGFR on the cell membrane.

### 3.6. Peptides 01cys_EGFR and 06cys_EGFR Did Not Affect the Cell Cycle

**Figure 7:**
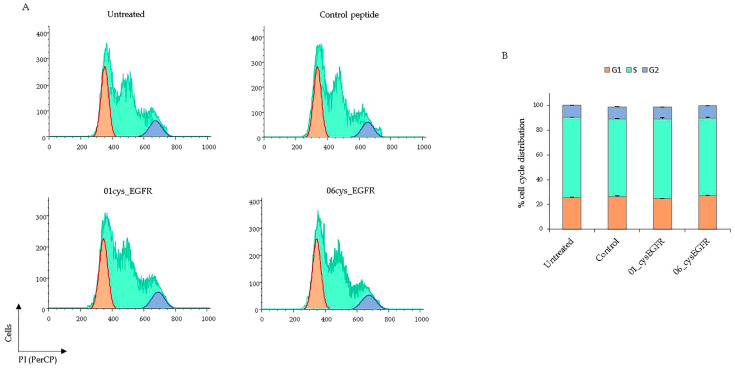
(**A**) The proportion of BT549 cells untreated or treated with 25 μM of control, 01cys_EGFR, and 06cys_EGFR peptides in G1, S, and G2 phases were determined by flow cytometry and quantified by Flow Jo software using Watson Pragmatic method. (**B**) Histograms showed the percentage of cell cycle phase distribution of PI-stained TNBC cells. Data are collected from two different experiments and presented as means ± SD. Paired *t*-test indicates no statistical significance between peptide-stimulated and unstimulated cells.

Next, we tested whether the peptides 01cys_EGFR and 06cys_EGFR could affect the cell cycle through the binding to EGFR. To this end, BT549 cells were serum starved for 18h to induce synchronization and then treated with 01cys_EGFR, 06cys_EGFR, and the irrelevant control peptides (25 μM) or left untreated for 24 h. Proliferating cells were stained with propidium iodide to analyze cell cycle distribution by flow cytometry (Figure 7A). The cells treated with 01cys_EGFR, 06cys_EGFR, and control peptides were distributed ~26% in G1, ~64% in S, and ~10% in the G2 phase, which was a similar behavior to unstimulated cells (Figure 7B). These data suggest that, in our experimental conditions, 01cys_EGFR and 06cys_EGFR peptides lacked mitogenic activity through the binding to the EGFR.

## 4. Discussion

TNBC is the most aggressive form of breast cancer. There are different TNBC subtypes characterized by intratumor heterogeneity based on morphology, gene expression profile, and response to adjuvant chemotherapies [7,8,9]. Unfortunately, TNBC is still lacking effective anticancer targets for clinical treatment [64,65]. This justifies the urgency to explore new biomarkers and therapeutic strategies for improving the survival outcomes of TNBC patients. EGFR is a tyrosine kinase membrane receptor that contains in the extracellular region the docking site for the epidermal growth factor and several other ligands that stimulate the intracellular signaling of cell growth and survival. As the EGFR is overexpressed in mesenchymal TNBC, which correlates with poor prognosis [17], it represents a potential tumor biomarker and target for personalized tumor therapy. In the last decade, several monoclonal antibodies and tyrosine kinase inhibitors have been developed for EGFR-targeted therapies [25,26]. However, they were revealed to be useless in TNBC patients. More recently, EGFR-targeting antibodies or peptides were developed as new tools to be conjugated to nanocarriers of anticancer drugs [27]. In particular, the peptide ligands are optimal tools for targeting membrane receptors on tumor cells, as they are smaller than antibodies, easily synthesized, and chemically modified in order to improve their stability, solubility, tissue penetration, and biocompatibility. Further, they are immunosafe and can be used to functionalize nanocarriers for tumor-targeted delivery of anticancer drugs. In this context, the phage display technology allows the identification of high-affinity peptide ligands for a specific target, such as membrane receptors that are overexpressed in cancer [28]. Several research groups have exploited the targeting of EGFR using specific peptide ligands selected by phage display in different tumor contexts. For example, linear 7-mer peptide ligands SYPIPDT and HTSDQTN were selected on A-431 epidermoid cancer cells and exhibited binding affinities for EGFR and dose-dependent inhibitory effects on EGFR protein phosphorylation [37]. The linear QRHKPRE peptide was Cy5.5- conjugated, validated for in vitro and in vivo binding affinity, and exploited for tumor imaging and photodynamic therapy in mouse colorectal cancer models [38,39]. Phage display was also used to identify peptide ligands of the tumor-specific EGFRvIII mutant form in several tumor cell lines [45]. The linear GE11 dodecapeptide YHWYGYTPQNVI was selected against the purified human EGFR protein and tested in EGFR-positive cancer cells and tumor xenografts [40] and was further conjugated to different drug delivery nanocarriers for targeting liver, ovarian and colorectal cancers [41,42,43]. In these studies, the peptides were selected using phage display libraries exposing linear peptides using the purified EGFR protein as bait, and then the synthetic analogues modified by computational studies were developed as EGFR-specific radioligands. For EGFR targeting on TNBC cells, the synthetic analogue of the GE11 peptide, named peptide 22 (YHWYGYTPENVI), showed the highest uptake to MDA-MB-468 and MDA-MB-231 cells but low serum stability [44]. Of interest, the cyclization of peptide 22 was necessary to improve its in vivo applicability.

In our study, we took into consideration the criticisms of previous studies, and thus we chose to screen a phage display library that exposed cyclic peptides using as bait the MDA-MB-231 cells, a TNBC cell line that overexpresses EGFR. We reasoned that whole cell-based screening was more suitable than the screening with the purified EGFR protein or the EGFR extracellular domain since the membrane receptor, when expressed on the cell surface, preserves its native folding and the post-translational modifications. The peptides 01_cysEGFR and 06_cysEGFR were selected for the highest affinity and specificity binding to EGFR-positive cells, as shown by FACS and confocal microscopy. Further, the peptide sequences were modeled and docked to the EGFR extracellular domain to evaluate the binding affinity and the type of interaction with the EGFR pocket. The dissociation constant of the 01_cysEGFR peptide was lower than the 06_cysEGFR peptide, suggesting its better affinity binding to EGFR. The results of bioinformatics analysis correlated with the binding of the FITC-conjugated peptides to human and murine EGFR-positive TNBC cells as measured by flow cytometry. Further, the ex vivo confocal analysis of TNBC xenografts confirmed the higher EGFR targeting ability of 01_cysEGFR peptide than that of 06_cysEGFR peptide, as shown by the co-localization of peptides with the EGFR receptor expressed in tumor tissues. Both 01_cysEGFR and 06_cysEGFR peptides did not affect cell division, as shown by cell cycle analysis in the presence and absence of the peptides. This behavior was consistent with the potential role of the peptides as targeting agents devoid of mitogenic activity and, thus, optimal tools for tumor targeting therapy. In conclusion, we have identified two peptide ligands of the EGFR that show high affinity and specificity binding to the EGFR overexpressed in TNBC cells, as shown by flow cytometry and confocal microscopy. In perspective, these new peptides could be used to functionalize nanocarriers for tumor-targeted delivery of anticancer drugs.

## 5. Conclusions

Our approach holds great promise for finding and characterizing novel peptides that target the EGFR tyrosine kinase membrane receptor overexpressed in TNBC. Conjugation of EGFR-specific peptides to the surface of anticancer drug-loaded nanoparticles could be a more effective therapeutic strategy focusing on drug delivery to mesenchymal TNBC tumor masses.

## Figures and Tables

**Table 1:** HADDOCK and PRODIGY results of 01_cys EGFR and 06_cys EGFR—EGFR complexes.

	HADDOCK Score *	Cluster Size	RMSD **	VdW E_vdw_	Electrostatic E_elec_	Desolvation Energy	Buried Surface Area	Z-Score ***
01_cysEGFR	−42.4 ± 5.7	86	0.4 ± 0.3	−37.5 ± 2.2	−111.7 ± 22.3	0.8 ± 2.2	1176.4 ± 56.0	−1.6
06_cys EGFR	−47.4 ± 1.3	28	1.6 ± 0.1	−33.2 ± 3.5	−198.4 ± 42.3	6.4 ± 0.6	1097.1 ± 37.4	−1.8
Binding Affinity calculated with PRODIGY server								
			Binding Affinity (ΔG) ^§^		Predicted Dissociation Constant (K_D_)		Temperature (°C)	
01_cysEGFR			−10.1		7.7 × 10^−8^		37	
06_cysEGFR			−8.5		1.1 × 10^−6^			

* The HADDOCK score is defined as: 1.0 Evdw + 0.2 Eelec + 1.0 Edesol + 0.1 EAIR. ** Root Main Square Deviations (RMSD) from the overall lowest-energy structure. *** HADDOCK Z-score indicates standard deviations from the average cluster (the more negative, the better). ^§^ Value expresses in kcal mol^−1^.

## Data Availability

The data presented in this study are available on request from the corresponding authors.

## Supplementary Materials

The following supporting information can be downloaded at: https://www.mdpi.com/article/10.3390/cells12071078/s1, Figure S1. Phage enrichment during EGFR phage-display screening; Figure S2. Analysis of phage binding ability to MDA-MB-231 cells using flow cytometry; Figure S3. Peptide binding assay in EGFR-positive thyroid cancer cells using flow cytometry.
