# Supplementary material for: Development of Cyclic Peptides Targeting the Epidermal Growth Factor Receptor in Mesenchymal Triple-Negative Breast Cancer Subtype
Source: Cells. 2023 Apr 3;12(7):1078. doi: 10.3390/cells12071078 (PMC10093212; doi:10.3390/cells12071078)
Supplement: Supplementary file 1 [file cells-12-01078-s001.zip › cells-2259763-supplementary.pdf]

Supplementary Figures

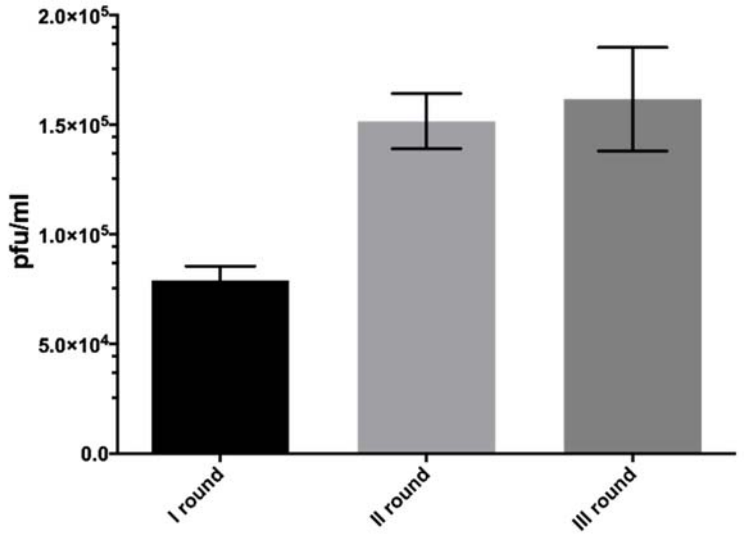

Figure S1. Phage enrichment during EGFR phage-display screening. Titers of eluted phages after each round of biopanning were plotted as pfu/mL. A progressive increase in the colony-forming capacity of EGFR bound clones was observed during first (8×10^4 pfu/mL), second (1.5×10^5 pfu/mL) and third round (1.6×10^5 pfu/mL) of bio panning.

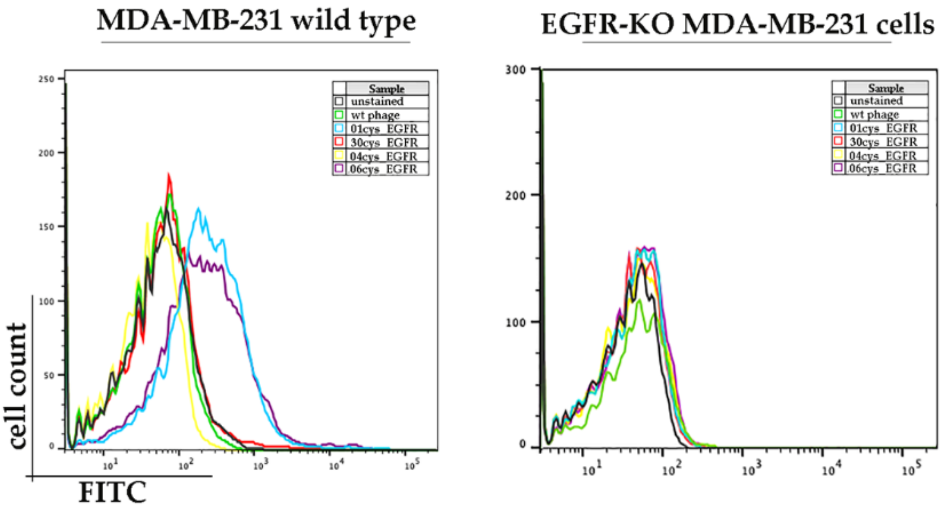

Figure S2. Analysis of phage binding ability to MDA-MB-231 cells using flow cytometry. FACS analysis of binding ability of FITC- conjugate phages 01cysEGFR, 04cys_EGFR, 06cys_EGFR and 30cys_EGFR on wild type MDA-MB-231 cells compared to EGFR KO MDA-MB-231. Wild type M13 phage was used as a negative control.

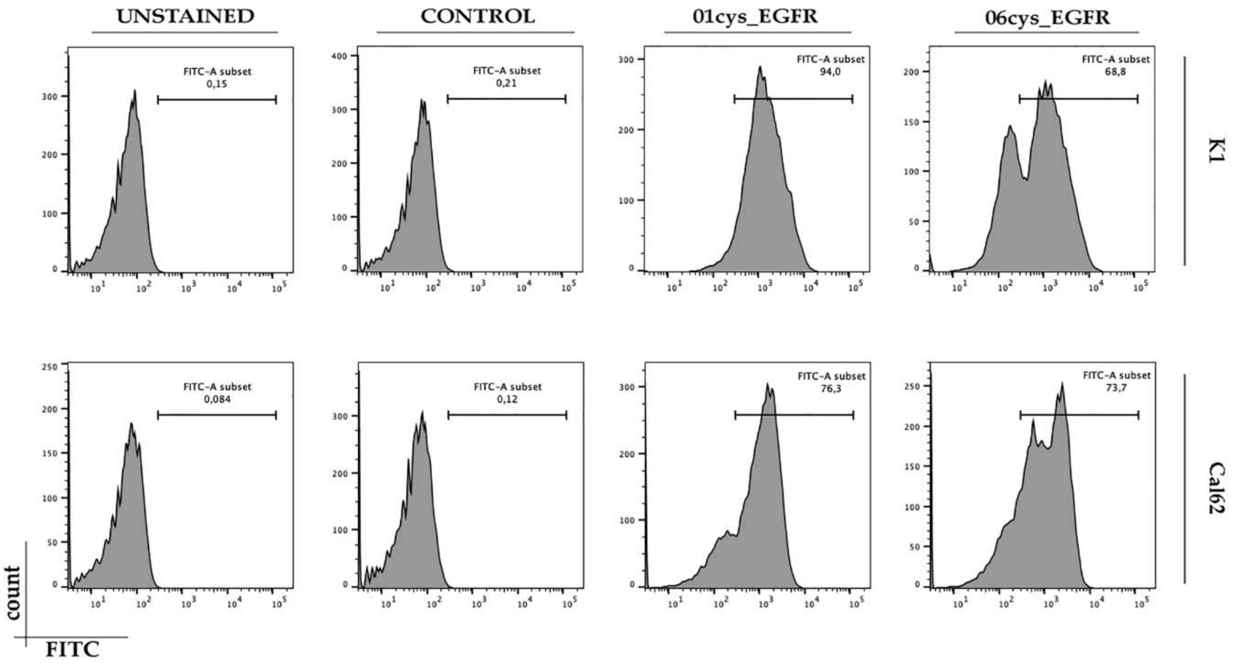

Figure S3. Peptide binding assay in EGFR-positive thyroid cancer cells using flow cytometry. Flow cytometric analysis showing EGFR-overexpressing thyroid cancer cell lines K1 and Cal62 unstained or stained with 10uM of FITC- conjugate peptides (control, 01_cysEGFR and 06cys_EGFR) for 45 min.
